# Comparative Analysis of Complete Chloroplast Genomes of *Penstemon digitalis* Nutt. ex Sims and Insights into Phylogenetic Relationships of *Penstemon*

**DOI:** 10.3390/genes17101271

**Published:** 2026-10-13

**Authors:** Changmei Du, Yaqian Chen, Tingting Fang, Jianhua Yue, Shoufu Gong, Yan Dong

**Affiliations:** 1 School of Horticulture, Xinyang Agriculture and Forestry University, Xinyang 464000, China; yandcm@163.com (C.D.);; 2 School of Forestry, Xinyang Agriculture and Forestry University, Xinyang 464000, China

**Keywords:** *Penstemon digitalis*, chloroplast genome, comparative genomics, phylogenetic relationships, molecular markers, Plantaginaceae

## Abstract

**Background:** *Penstemon*, a core genus of the tribe Cheloneae in Plantaginaceae, possesses significant ecological and horticultural value. However, its interspecific phylogenetic relationships remain unclear due to morphological convergence, hybridization events, and limited genomic resources. This study aimed to characterize the complete chloroplast (cp) genome of *Penstemon digitalis* and explore its genomic features and phylogenetic position within Plantaginaceae. **Methods:** We sequenced the cp genome of *P. digitalis* using high-throughput sequencing, assembled and annotated the genome, and compared it with other Plantaginaceae species. Repetitive sequences and hypervariable regions were identified. Phylogenetic analyses were performed using maximum likelihood (ML) based on complete cp genomes from 46 taxa (36 Plantaginaceae, six Scrophulariaceae, and four outgroup species). **Results:** The cp genome is 152,993 bp with a typical quadripartite structure, containing 132 functional genes (87 protein-coding, 37 tRNA, and eight rRNA). Comparative analyses showed high gene content and structural conservation within the tribe Cheloneae. Several hypervariable regions and repeats were detected as potential markers. ML analysis strongly supported the monophyly of *Penstemon* among the sampled species (100% bootstrap) and placed *P. digitalis* in a well-supported clade within the genus; *Penstemon* was recovered as the sister group of *Russelia equisetiformis*. **Conclusions:** This study provides the first complete cp genome resource for *P. digitalis*, confirming its phylogenetic affinity and offering valuable genomic data for future studies on population genetics, species delimitation, and adaptive evolution in *Penstemon*. These findings will aid taxonomic revision and conservation efforts for this diverse genus.

## 1. Introduction

*Penstemon* is one of the largest and most diverse plant genera in North and Central America, encompassing over 280 species [1,2]. Species in this genus display remarkable morphological diversity and have successfully colonized a wide range of environments, giving them great potential for landscaping and ecological restoration. Their adaptability implies vast possibilities for enhancing the aesthetic appeal of various landscapes, and many species have been introduced into cultivation as ornamental perennials [3,4]. Among them, *Penstemon digitalis* Nutt. ex Sims is an evergreen herbaceous perennial reaching 60–100 cm in height. The leaves are opposite, with entire margins, and are oblong to broadly lanceolate. The inflorescence is racemose, and the flowers are predominantly white with variable purple striping; glandular trichomes cover the flowers and stems. The flowers are bisexual, and the species blooms from spring to summer [5]. Owing to its compact habit and elegant flowers, this species can be grown at forest edges, along walls or roadsides, and in flower borders, making it a highly valuable ornamental plant. In addition, *P. digitalis* contains phenylethanoid glycosides with neuroprotective, anti-inflammatory, and antioxidant activities, giving the species potential medicinal value in the treatment of neurodegenerative diseases [6].

In the traditional classification system, the genus *Penstemon* was usually placed within the family Scrophulariaceae. However, subsequent studies have revealed that this family is polyphyletic, leading to considerable confusion in the branching and classification of its species [7]. Albach [8] reclassified this genus into the family Plantaginaceae based on molecular evidence. Nevertheless, there remains a paucity of comprehensive molecular data to firmly establish and support the classification of this genus, particularly regarding the interspecific relationships within *Penstemon*, which have long been obscured by morphological convergence and putative hybridization events.

Among the three plant genomes, the chloroplast (cp) genome is characterized by a high degree of conservation, maternal inheritance, and abundant genetic information and has therefore been widely used in studies of species evolution, phylogenetic relationships, and genetic diversity [9,10,11]. Relative to the nuclear and mitochondrial genomes, the cp genome offers a favorable balance between evolutionary rate and structural stability, making it particularly suitable for resolving relationships at both higher and lower taxonomic levels. However, to the best of our understanding, only seven *Penstemon* species had reported cp genomes before this study. Given the large size of the genus (ca. 280 species), this represents a severe sampling gap that hinders comprehensive evolutionary and taxonomic studies, and there is thus a pressing need to expand the genomic resources available for the genus. In the present study, we characterized the cp genome of *P. digitalis* with three objectives: (1) to provide a detailed description of its genomic features; (2) to determine the phylogenetic position of the species within Plantaginaceae; and (3) to lay a foundation for future genetic and evolutionary studies of *Penstemon*, including population genetics, species delimitation, and adaptive radiation.

## 2. Materials and Methods

### 2.1. Plant Materials and DNA Extraction

Plant material of *P. digitalis* was collected from Baihua Garden (114°12′ E, 32°16′ N, altitude: 102 m), Xinyang City, Henan Province, China. Professor Jianhua Yue from Xinyang Agriculture and Forestry University authenticated the plant material. The voucher specimen (code: PDN20230825) was deposited at the Herbarium of the Horticultural Plant Biotechnology Laboratory, Xinyang Agriculture and Forestry University. Fresh leaves were wrapped in aluminum foil, frozen in liquid nitrogen, and sent to Shanghai Origingene Biotechnology Co., Ltd (Shanghai, China)., for whole-genome sequencing. Total DNA of *P. digitalis* was extracted using a HiPure SF Plant DNA Mini Kit (Magen, Guangzhou, China). DNA concentration and quality were assessed using a NanoDrop 2000 spectrophotometer (Thermo Fisher Scientific, Waltham, MA, USA) and 1% (*w*/*v*) agarose gel electrophoresis with an Owl™ A2 horizontal electrophoresis system (Thermo Fisher Scientific, Waltham, MA, USA), respectively.

### 2.2. Genome Sequencing, Assembly and Annotation

Libraries with an average insert size of 500 bp were constructed from the DNA of *P. digitalis* and sequenced on the Illumina NovaSeq 6000 platform (Illumina, San Diego, CA, USA) following the standard protocol. Adapters and low-quality reads were removed from the raw data, and the quality of the filtered reads was evaluated with FastQC v0.11.4 [12]. De novo assembly of the cp genome was performed with GetOrganelle v1.7.0+ [13]. The Plastid Genome Annotator (PGA) (https://github.com/quxiaojian/PGA, accessed on 30 October 2023) [14] and GeSeq v.2.03 (https://chlorobox.mpimp-golm.mpg.de/geseq.html, accessed on 30 October 2023) [15] were used for sequence annotation, and the annotation results were manually corrected by cross-checking the PGA and GeSeq outputs: gene boundaries, start/stop codons, and intron/exon structures were compared against those of closely related Plantaginaceae plastomes and adjusted where discrepancies were found. The annotated cp genome sequence was submitted to the National Center for Biotechnology Information (NCBI) database under GenBank accession number NC_086665.1. The circular cp genome map was drawn with the online tool OGDRAW [16] (https://chlorobox.mpimp-golm.mpg.de/OGDraw.html, accessed on 10 September 2025).

### 2.3. Repeat Structure Analysis

Repeat sequences (palindromic, reverse, forward, and complementary) were detected with REPuter v1.0 [17] (https://bibiserv.cebitec.unibielefeld.de/reputer, accessed on 3 August 2025), using a Hamming distance of 3, a maximum computed repeat length of 5000 bp, and a minimum repeat size of 30 bp. Simple sequence repeats (SSRs) were identified with MIcroSAtellite (MISA) v1.0 [18] (https://webblast.ipk-gatersleben.de/misa/index.php, accessed on 3 August 2025 ), with thresholds of 10, 6, 5, 5, 5, and 5 for mono- to hexanucleotide motifs, respectively, and a minimum distance of 100 bp between adjacent SSRs.

### 2.4. Comparative Genome Analysis

Relative synonymous codon usage (RSCU) for cp genomes was computed with CodonW v1.4.4 [19] (https://galaxy.pasteur.fr/?form=codonw, accessed on 13 April 2025). A codon was considered preferred when RSCU > 1, whereas RSCU = 1 indicated no bias [20,21]. Codon usage distributions of the 10 species were visualized as a heatmap based on RSCU values using TBtools-II v2.149 [22]. The complete cp genomes of *P. digitalis* and nine other Plantaginaceae species were aligned with MAFFT v7.158b (Multiple Alignment using Fast Fourier Transform) [23], and sequence differences were visualized with mVISTA (http://genome.lbl.gov/vista/mvista/submit.shtml, accessed on 3 August 2025) [24] in shuffle-LAGAN mode [25]. Nucleotide diversity (Pi) was calculated in DnaSP v6.12.03using a sliding-window analysis with a window length of 400 bp and a step size of 200 bp [26]. Candidate hypervariable regions were defined as windows with Pi ≥ 0.10 located at local peaks of the Pi profile, irrespective of coding or non-coding regions; these regions were considered potential molecular markers. In addition, IRscope (https://irscope.shinyapps.io/irapp/; accessed on 5 December 2025) was used to evaluate the expansion and contraction of the inverted repeat (IR) regions with the GenBank files [27].

### 2.5. Phylogenetic Analysis

Complete cp genomes of 46 species (*P. digitalis*, 35 additional Plantaginaceae species, six Scrophulariaceae species, and four outgroup taxa from Lamiaceae, Lythraceae, Gesneriaceae, and Oleaceae) were retrieved from NCBI. Phylogenetic analysis was performed as follows: sequences were aligned with MAFFT v7.158b [23], conserved blocks were retained using Gblocks v0.91b [28], and the optimal substitution model (GTR+G) was selected with jModelTest v2.1.7 [29]. The maximum likelihood tree was then constructed with RAxML v8.2.12 [30] under the GTRGAMMA model, and nodal support was assessed with 1000 bootstrap replicates.

## 3. Results

### 3.1. Chloroplast Genome Characteristics Analysis

**Figure 1:**
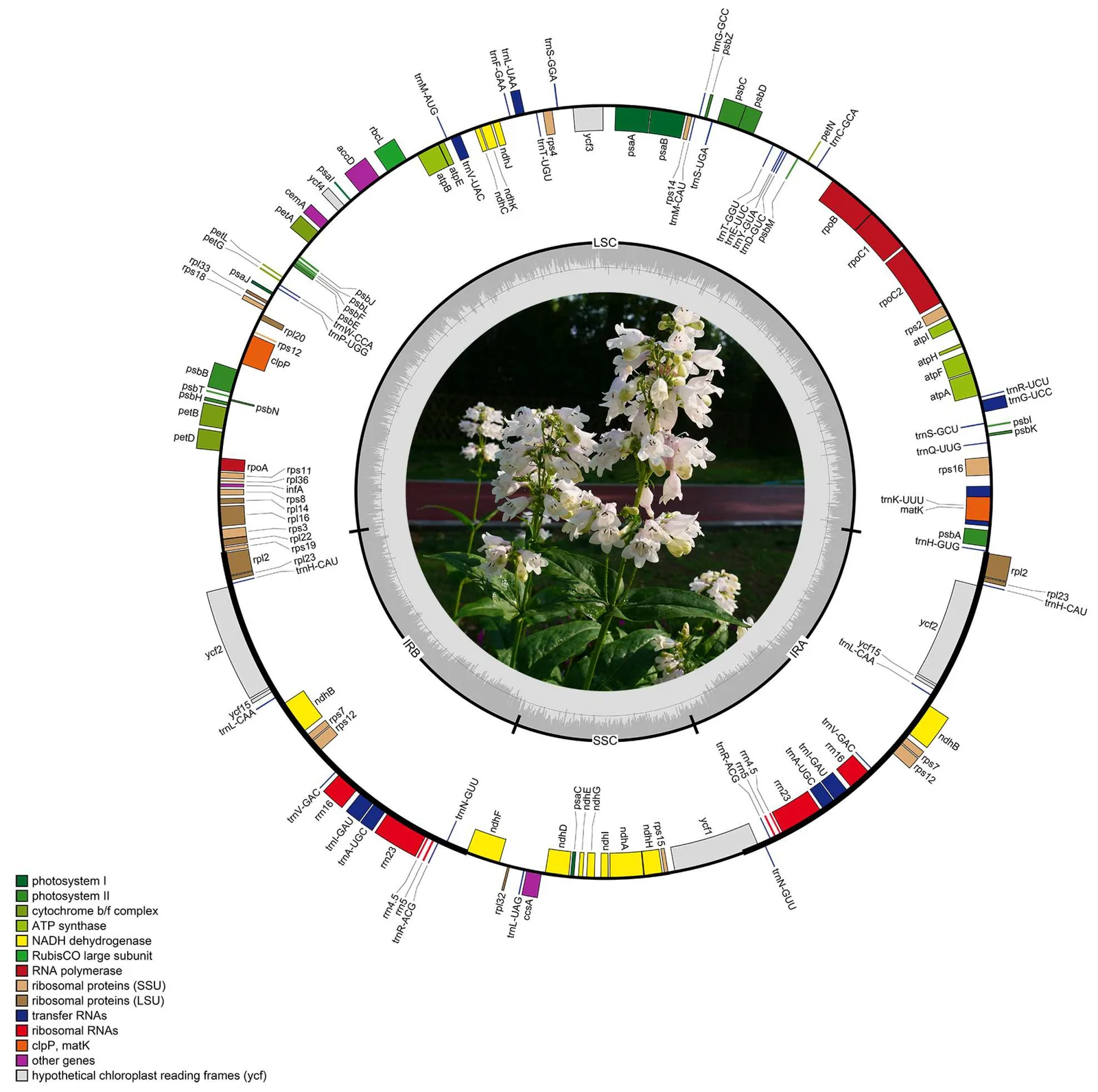
The chloroplast genome map of *P. digitalis*. Forward-coding genes are positioned on the outer circle, while reverse-coding genes are on the inner circle. Gray circles represent GC content.

In total, 58.01 million raw reads were generated, and 57.96 million clean reads (8.63 Gb) were retained after quality filtering, with Q20 and Q30 values of 97.44% and 92.81%, respectively, and a GC content of 35.63%. The clean reads were assembled by GetOrgnelle v1.7.0+ into a single circular contig corresponding to the cp genome, with an average sequencing depth of 878.59× (Figure S1). The *P. digitalis* cp genome exhibited a typical quadripartite structure (Figure 1), comprising two IR regions, a large single-copy region (LSC), and a small single-copy region (SSC), with the two single-copy regions separated by the IR regions. The genome was 152,993 bp in size with an overall GC content of 37.85%. The LSC, SSC, and IR regions were 84,157 bp, 17,768 bp, and 25,534 bp in length, with GC contents of 35.90%, 31.79%, and 43.19%, respectively. The GC content of the IR region was markedly higher than that of the SSC and LSC regions.

The cp genome of *P. digitalis* contains 132 genes, comprising 87 protein-coding genes (PCGs), 37 tRNA genes, and eight rRNA genes (Figure 1; Table 1). By function, these genes fall into four categories: 74 involved in self-replication, 45 related to photosynthesis, the *ycf* genes, and 13 classified as “other”. Among them, 114 genes are present as single copies, and 18 genes are located in the IR regions. In addition, 18 genes contain introns, of which 15 (*atpF*, *ndhA*, *ndhB*, *petB*, *petD*, *rpl16*, *rpl2*, *rpoC1*, *rps16*, *trnA-UGC*, *trnG-UCC*, *trnI-GAU*, *trnK-UUU*, *trnL-UAA*, *trnV-UAC*) have two exons, while the remaining three (*clpP*, *rps12*, *ycf3*) have three exons.

### 3.2. Distribution of SSRs and Long Repeat Sequences

**Figure 2:**
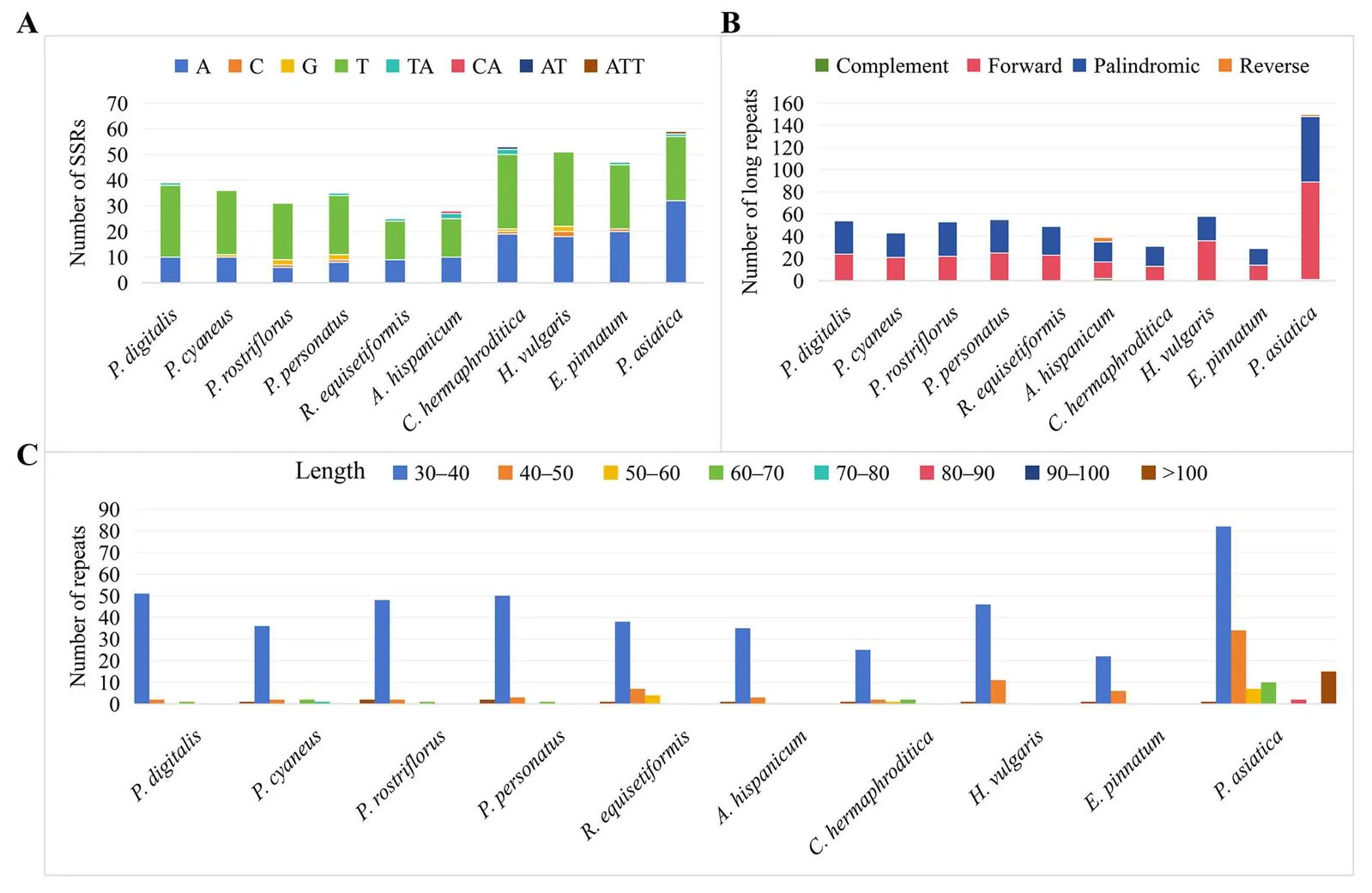
Analysis of repeat sequences in the cp genomes of 10 species in the Plantaginaceae family. (**A**) Numbers of SSRs by type. (**B**) Numbers of long repeats by type. (**C**) Numbers of repeats by length class. Note: *P. digitalis* = *Penstemon digitalis*; *P. asiatica* = *Plantago asiatica*.

We characterized SSRs in the cp genomes of 10 Plantaginaceae species and found that the number of SSRs ranged from 25 (*Russelia equisetiformis*) to 59 (*Plantago asiatica*) (Figure 2A). In terms of type, mononucleotide SSRs accounted for the highest proportion, followed by dinucleotide SSRs, and only *Plantago asiatica* contained a single trinucleotide SSR. A/T repeats were the dominant type, accounting for 96.53% of all SSRs, and no tetranucleotide, pentanucleotide, or hexanucleotide SSRs were detected in any of the species.

To further examine repeat dynamics within the family, we compared long repeats across the 10 cp genomes (Figure 2B,C). Four types of long repeats were detected: forward, reverse, complementary, and palindromic. The total number of long repeats per species ranged from 29 to 150, with forward (13–88) and palindromic (15–59) repeats predominating. Complementary repeats occurred only in *Antirrhinum hispanicum* (two) and *Plantago asiatica* (one), whereas reverse repeats were recorded in *P. digitalis* (one), *R. equisetiformis* (one), *A. hispanicum* (four), and *P. asiatica* (two), indicating clear species specificity. Repeats of 30–40 bp were the most frequent.

### 3.3. Characteristics of Codon Usage

**Figure 3:**
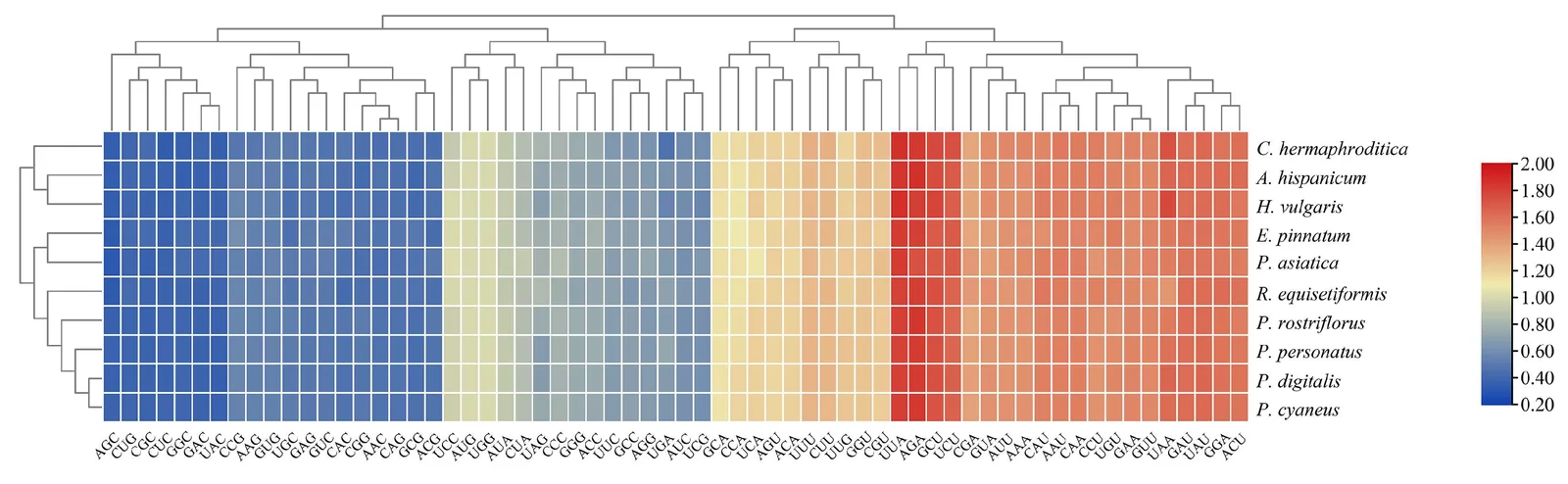
Heatmap distribution of codon usage in 10 plant species of the Plantaginaceae family. Horizontal axis: different codons. Vertical axis: different species. The cluster dendrograms on the left side and at the top of the heatmap show the clustering relationships of different species. Color indication: red represents high usage frequency, and blue represents low usage frequency. Note: *P. digitalis* = *Penstemon digitalis*; *P. asiatica* = *Plantago asiatica*.

**Figure 4:**
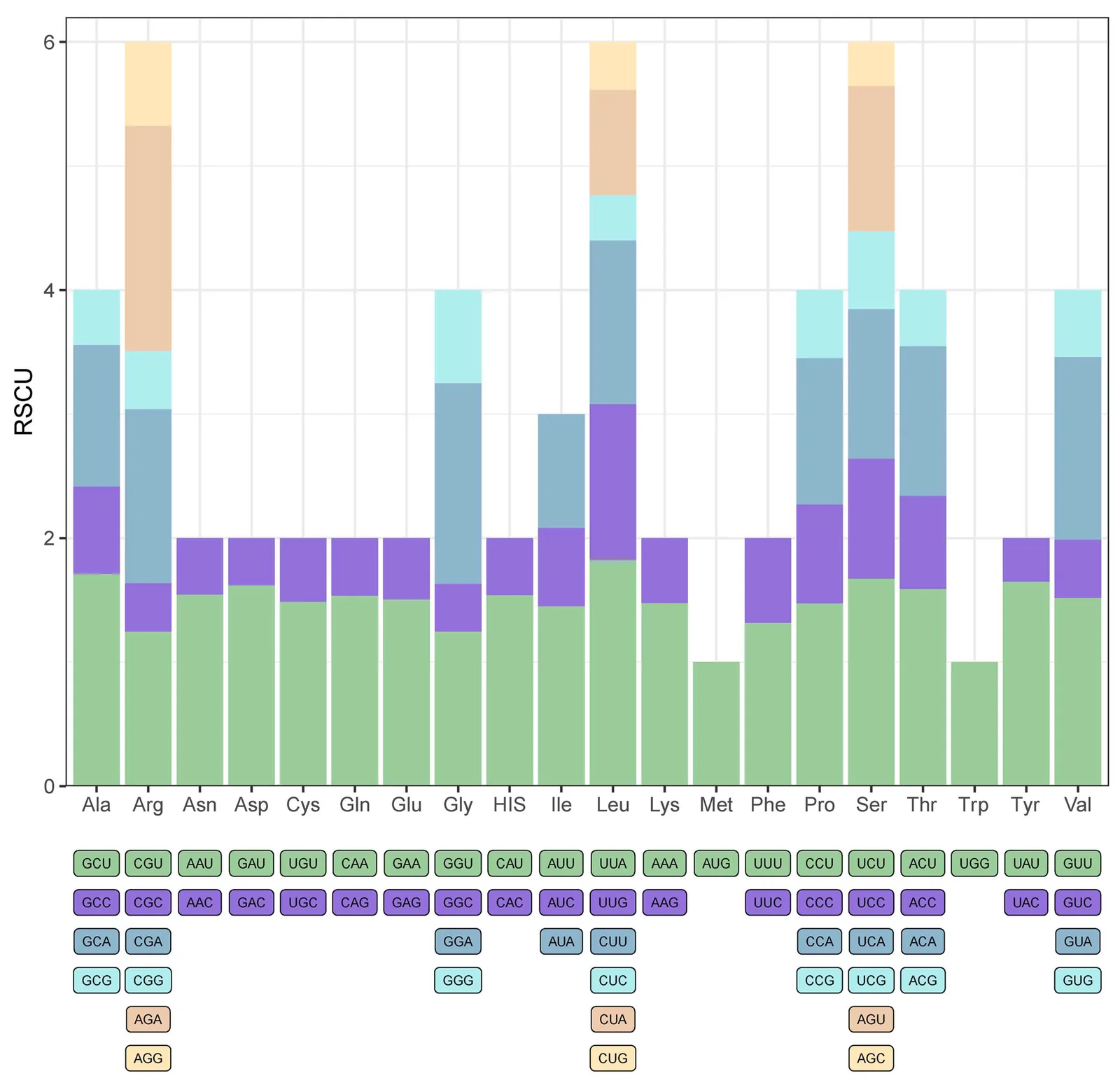
Codon usage mode of chloroplast genes in *P. digitalis*. Boxes below the graphs represent all codons encoding each amino acid, with the colors of the histograms corresponding to the colors of the codons.

The protein-coding genes in the complete cp genome of *P. digitalis* contain 24,710 codons. Analysis of codon usage in the cp genomes of the 10 Plantaginaceae species revealed a clear bias. The heatmap showed differences in codon usage frequency among species, although the overall patterns were highly similar (Figure 3). Codons UUU, AUU, AAU, AAA, GAU, and GAA were the most frequently used, whereas UGC, CGC, UAG, UAA, and UGA were the least frequent (Figure 4).

The RSCU provides an intuitive measure of codon usage bias. Of the 64 codons, 61 encode the 20 amino acids and three serve as stop signals; codon bias was evident for all amino acids except those encoded by a single codon (Trp and Met) (Table S1). In *P. digitalis*, 30 preferred codons were identified, covering 18 amino acids and one stop codon. The GC content at the third codon position was 25.29%, reflecting a strong A/T bias.

### 3.4. Contraction and Expansion of the IR Regions

**Figure 5:**
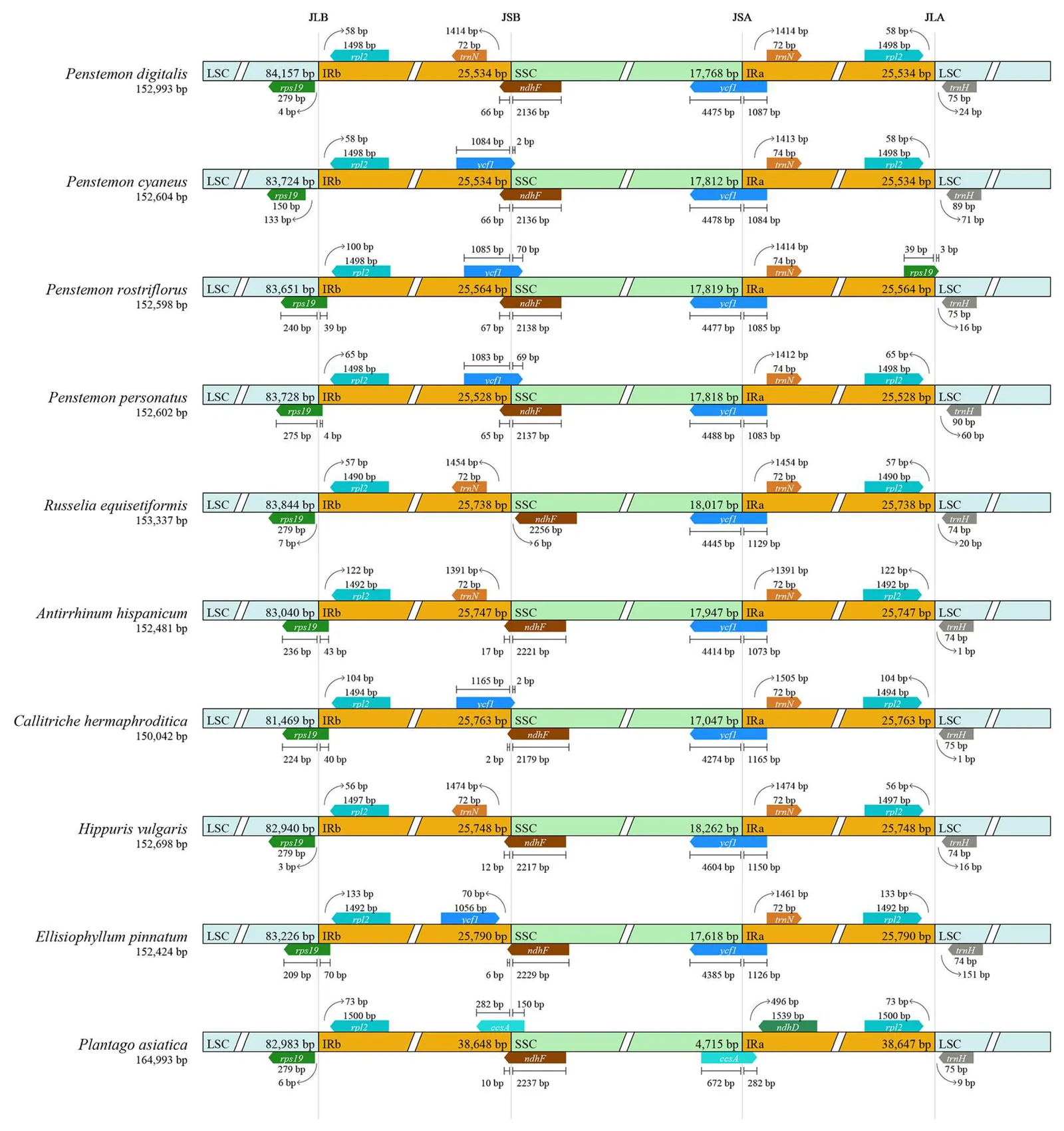
Comparison of the boundary positions of LSC, SSC, and IR regions in the cp genomes of 10 species in the Plantaginaceae family. JLB: junction line between LSC and IRb; JSB: junction line between IRb and SSC; JSA: junction line between SSC and IRa; JLA: junction line between IRa and LSC.

In the cp genome, the IR region is generally regarded as the most conserved area. We compared the junction positions of the LSC, SSC and IR regions in 10 species (Figure 5). The results showed that the positions and lengths of *rps19*, *rpl2*, *ycf1*, *trnN*, and *ndhF* at the four junctions varied among species. The variations among species of the *Penstemon* genus were relatively small, with minimal differences in the length of the IR region. At the JSB boundary, *P. digitalis* lacked *ycf1*, and the length of *ycf1* differed by 1 bp among the remaining species, while the length of *ndhF* differed by 1–2 bp. At the JSA boundary, the length of *ycf1* differed by 1–13 bp. At the JLA boundary, the length of *rpl2* was consistent, and at the JLB boundary, *rps19* showed relatively obvious changes in position and length. Overall, the differences between *Penstemon* and other genera were greater than those among *Penstemon* species.

### 3.5. Comparative Analysis of Complete Chloroplast Genomes

**Figure 6:**
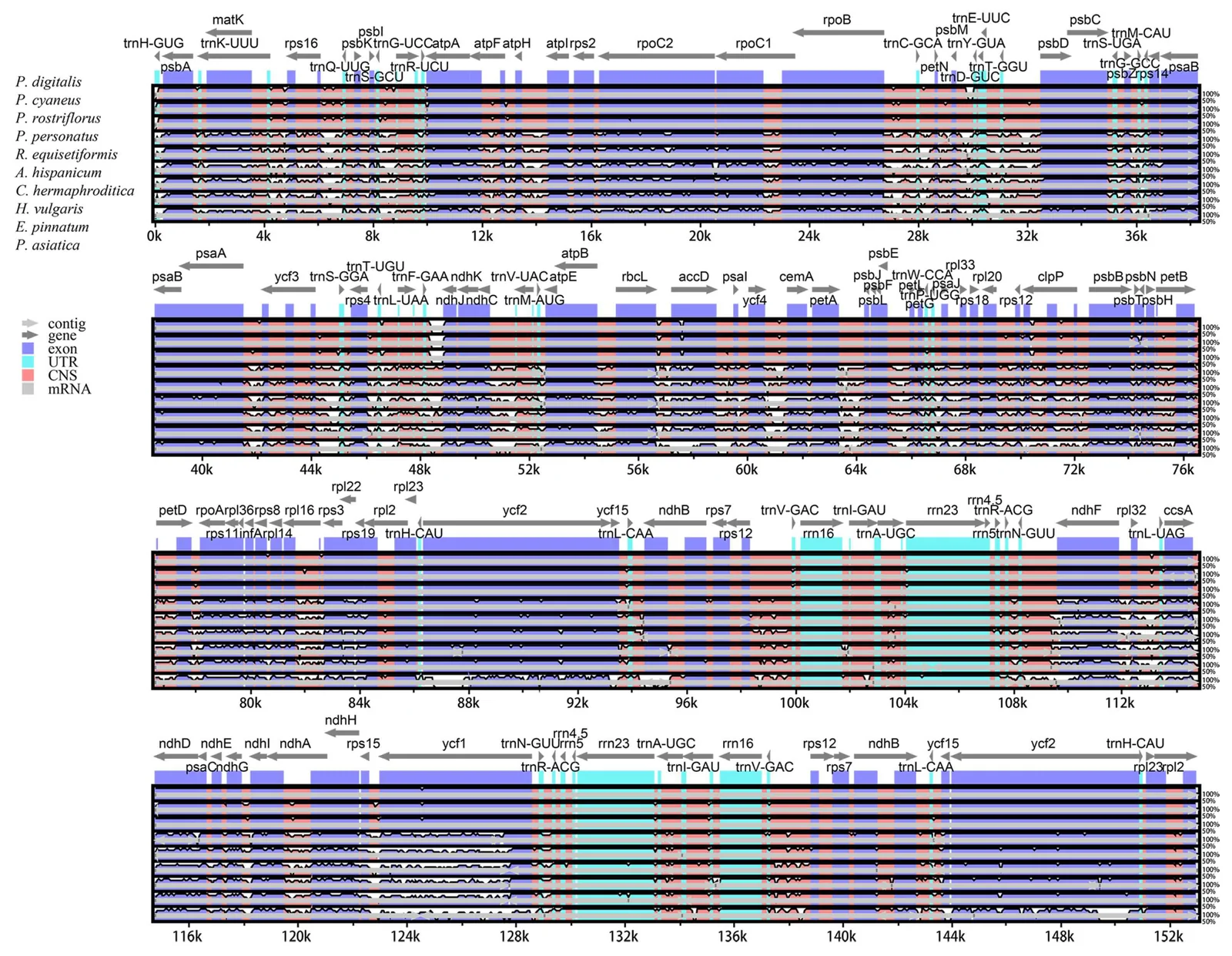
Comparison of cp genome sequences of 10 species in the Plantaginaceae family. Horizontal axis: coordinates along the cp genome; vertical axis: sequence similarity (50–100%). Arrows indicate genes and their direction of transcription. Genome regions are color-coded as protein-coding sequences, rRNA, tRNA, introns, and conserved non-coding sequences. Note: *P. digitalis* = *Penstemon digitalis*; *P. asiatica* = *Plantago asiatica*.

Sequence identity among the 10 Plantaginaceae species was analyzed with mVISTA, using the cp genome of *P. digitalis* as the reference (Figure 6). Across the aligned genomes, the IR regions were more conserved than the single-copy regions, and the coding regions were more conserved than the non-coding (intergenic) regions.

**Figure 7:**
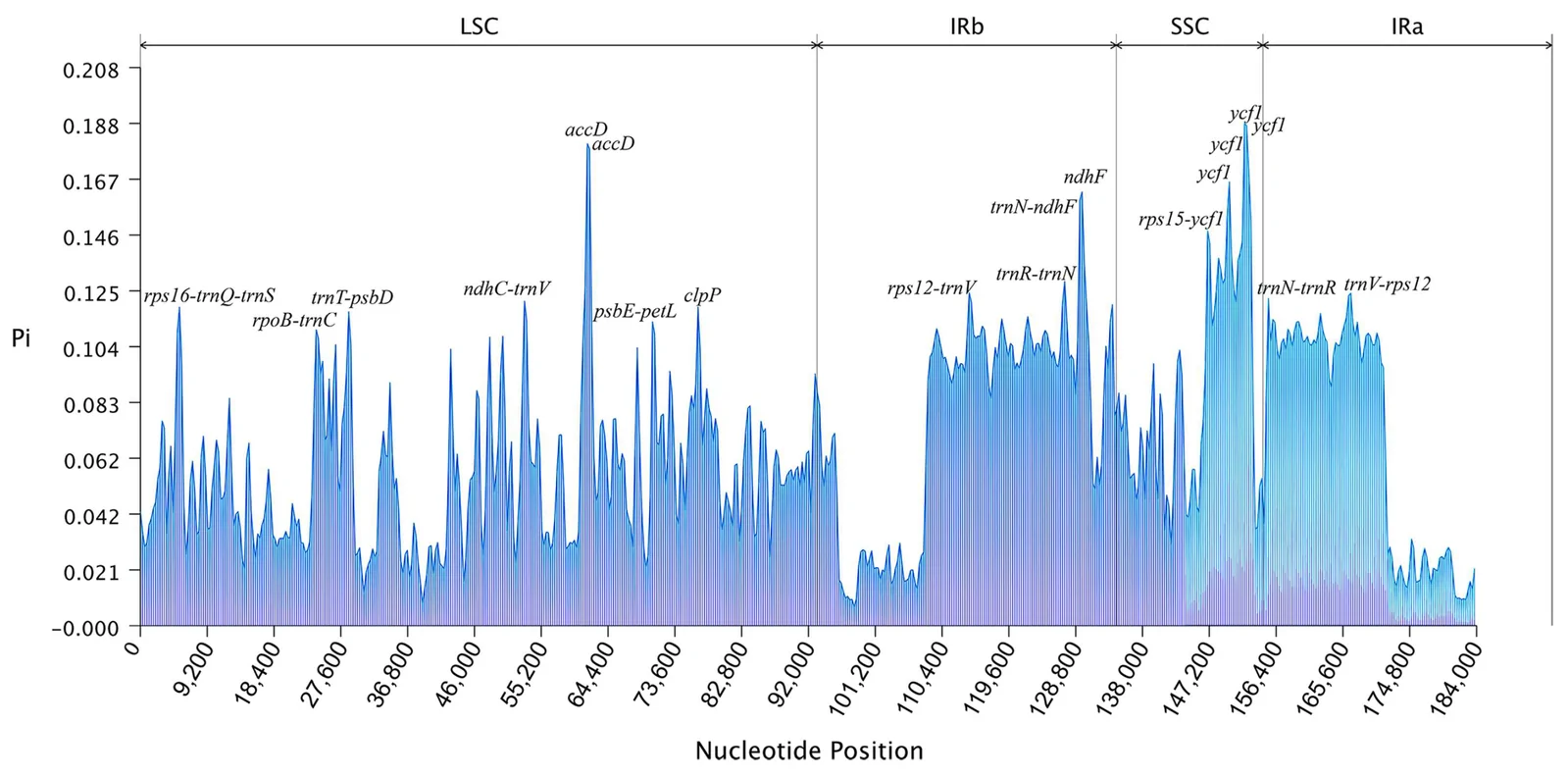
Nucleotide diversity (Pi) across the aligned plastomes of *P. digitalis* and related species, calculated in a sliding-window analysis (window length: 400 bp; step size: 200 bp).

Combined with the graph of Pi calculation (Figure 7), genes *accD*, *clpP*, *ndhF*, *rps15*, and *ycf1* showed relatively high sequence differences in the coding regions. Non-coding regions included *psbE*-*petL*, *rps16*-*trnQ*-*trnS*, *rpoB*-*trnC*, *trnT*-*psbD*, *ndhC*-*trnV*, *rps12*-*trnV*, *trnR*-*trnN*, and *trnN*-*ndhF*. All of these regions showed Pi values above 0.10 in the sliding-window analysis and were therefore considered candidate molecular markers; their practical utility should be verified in future phylogenetic studies of Plantaginaceae species. Additionally, sequences of *Penstemon* species were more similar to one another than to those of other genera.

### 3.6. Phylogenetic Relationships

**Figure 8:**
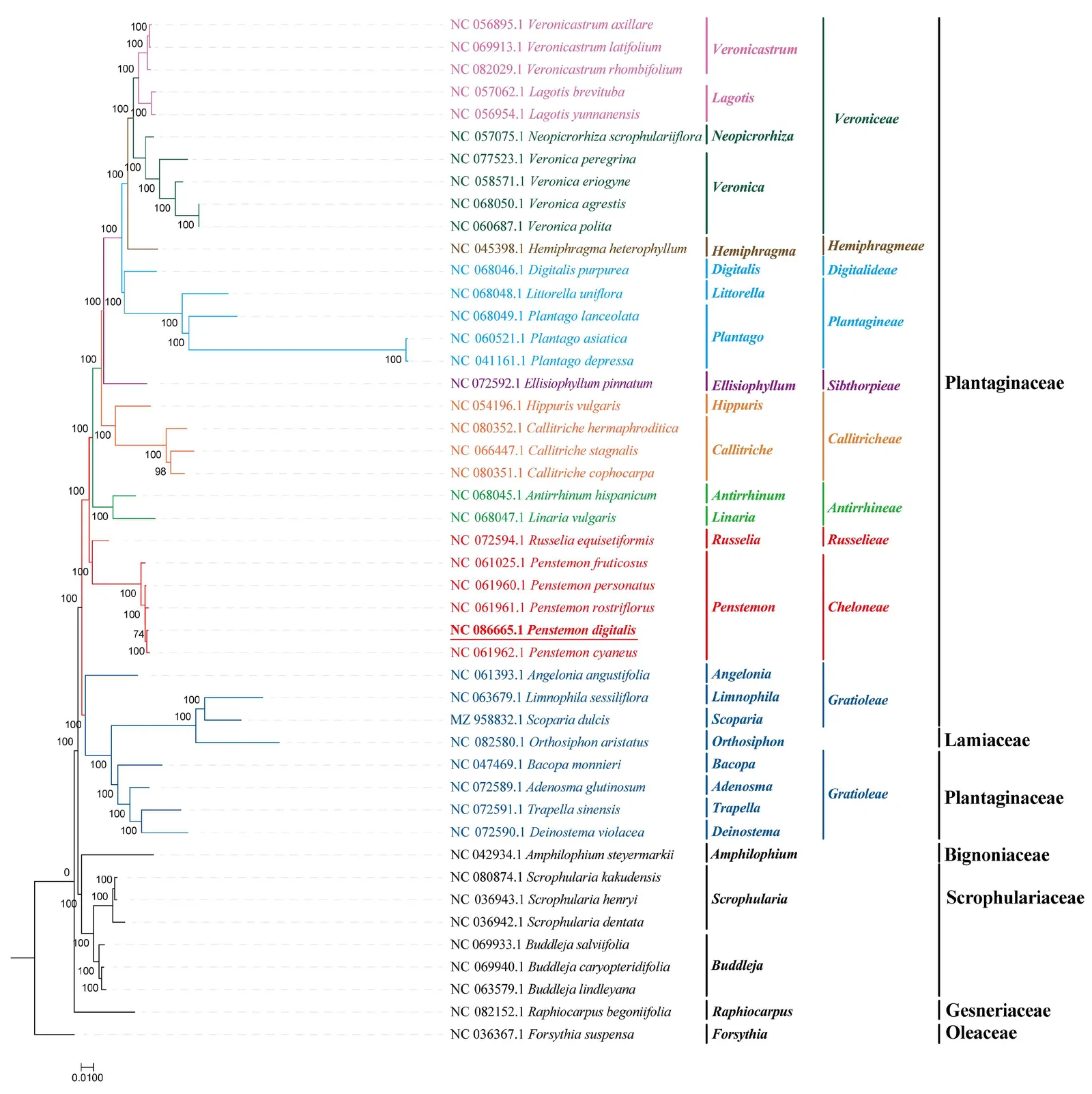
Maximum likelihood (ML) phylogenetic tree based on whole-chloroplast genome data, showing the phylogenetic position of *P. digitalis*; the numbers above the branches indicate ML bootstrap support values. The underlined species name indicates the species investigated in this study.

Phylogenetic analysis of *P. digitalis* and 45 other species showed that Plantaginaceae and Scrophulariaceae formed two distinct clades (Figure 8). Within Plantaginaceae, *Angelonia*, *Limnophila*, *Scoparia*, *Bacopa*, *Adenosma*, *Trapella*, and *Deinostema* clustered into one clade; whereas *Veronicastrum*, *Lagotis*, *Neopicrorhiza*, *Veronica*, *Hemiphragma*, *Digitalis*, *Littorella*, *Plantago*, *Elisiophyllum*, *Hippuris*, *Callitriche*, *Antirrhinum*, *Linaria*, *Russelia*, and *Penstemon* formed another. Notably, *Russelia* and *Penstemon* were grouped together with 100% bootstrap support, and the placement of *P. digitalis* within the genus was likewise supported at 100% bootstrap.

## 4. Discussion

The complete cp genome of *P. digitalis* shows the typical quadripartite architecture (LSC, SSC, and two IR regions) that is characteristic of most angiosperms, including its close relatives in Plantaginaceae [31,32]. Its size, gene content (132 genes, comprising 87 PCGs, 37 tRNAs, and eight rRNAs), and GC-content pattern (elevated in the IR regions) conform to the conserved features of angiosperm plastomes, indicating that *P. digitalis* has retained the genomic stability inherited from ancestral angiosperms [33,34,35,36]. This conservation is especially evident in genes involved in photosynthesis and self-replication, such as *psbA* (encoding the core protein of Photosystem II), *rbcL* (encoding the large subunit of ribulose-1,5-bisphosphate carboxylase), *rpoB* (encoding the β subunit of RNA polymerase) and *trnL* (a transfer RNA gene). These genes are central to energy conversion and information flow in the chloroplast: *psbA* and *rbcL* participate directly in photosynthetic electron transport and carbon fixation, the core reactions through which plants synthesize organic matter from light energy [37,38], whereas *rpoB* and *trnL* underpin the chloroplast’s own transcription and translation system and thereby ensure the faithful transmission of genetic information [39,40,41]. Because the integrity of these genes is essential to basic cellular function [42,43], they have experienced strong purifying selection over long-term evolution, which has preserved ancestral gene features, limited the accumulation of deleterious mutations, and produced the marked conservation observed today [44,45,46]. Likewise, the pronounced A/T bias at the third codon position (GC content of 25.29%) and the overall similarity of codon usage among the sampled species reflect the compositional constraints typical of plastomes and further underscore the conserved nature of Plantaginaceae cp genomes.

The IR region plays an important role in protecting gene structure and serves as an effective indicator for evaluating plant genetic diversity. However, the expansion or contraction of this region can lead to gene loss or gain, resulting in variations in cp genome size [47,48]. The comparison of IR boundaries revealed only minor variation among *Penstemon* species and somewhat greater differences relative to other sampled genera of Plantaginaceae. Such boundary variation tends to be phylogenetically structured, with closely related species generally showing more similar IR boundaries. However, the mechanisms underlying IR expansion and contraction are complex, and the present data do not justify attributing these differences directly to environmental adaptation or selective pressure; testing such causal hypotheses will require broader taxon sampling and explicit population-genetic or selection analyses.

Chloroplast SSRs are valuable tools for phylogenetic reconstruction and population genetic studies [49]. Comparative analysis of repeat sequences offers a useful window into the genetic diversity of Plantaginaceae. The abundance of A/T-rich SSRs in *P. digitalis* and other sampled species (96.53% of total SSRs) reflects a common bias toward AT nucleotides in cp genomes, likely driven by maternal inheritance and high mutation rates in non-coding regions [50,51]. These SSRs, together with the long repeats identified (predominantly forward and palindromic), represent candidate molecular markers which, after experimental validation at the population level, could enable fine-scale analyses of genetic structure and divergence within *Penstemon* and related genera. In particular, the high proportion of forward and palindromic repeats suggests that these elements may contribute to genome rearrangement and the formation of secondary structures, which are important for cp genome stability and evolution [52,53,54]. The scarcity and species specificity of reverse and complementary repeats may reflect lineage-specific evolutionary histories [55,56], while the predominance of 30–40 bp repeats may promote local recombination and thereby contribute to structural variation such as IR expansion and contraction [57]. The SSR markers identified here are also potentially applicable to assessments of gene flow, population bottlenecks, and phylogeographic patterns in *Penstemon* species, which often have large but fragmented distribution ranges; however, empirical validation is still needed.

Sequence identity analyses (mVISTA) and Pi calculations further highlight regions of high variability, such as *accD*, *clpP*, *ndhF*, *ycf1*, and intergenic spacers like *rps16-trnQ-trnS* and *ndhC-trnV*. These hypervariable regions, which were defined as windows with Pi values exceeding 0.10 in the sliding-window analysis and diverge more rapidly than conserved coding regions, represent promising candidate markers for resolving relationships among closely related species in Plantaginaceae—especially within *Penstemon*, a genus with complex morphological diversity and unresolved taxonomic boundaries. However, their practical utility should be confirmed experimentally before these markers are applied. The identification of these hotspots is particularly timely, as it provides practical tools for future studies aiming to disentangle the phylogenetic relationships among closely related *Penstemon* species that are morphologically similar but genetically distinct.

As one of the core groups in the order Lamiales, Plantaginaceae has undergone a significant transformation in its taxonomic boundaries and phylogenetic relationships, shifting from “morphology-based classification” to “molecular evidence-driven reconstruction”. In traditional classification systems [58], Plantaginaceae contained only three genera and was placed in the order Plantaginales, while Scrophulariaceae, as a “large family”, included hundreds of genera with diverse morphological characteristics, leading to taxonomic confusion. The Angiosperm Phylogeny Group (APG) I system (1998) [59] first re-circumscribed Plantaginaceae based on molecular evidence from plastid DNA regions and nuclear ribosomal DNA (e.g., *rbcL*, *trnL-F*), incorporating most genera from the former Scrophulariaceae (such as *Antirrhinum* and *Digitalis*), the entire family Hippuridaceae, and groups like Callitrichaceae into Plantaginaceae, and formally placed the family in Lamiales. Subsequent systems [60,61,62,63] gradually optimized this framework, with APG III defining 12 tribes within Plantaginaceae—a classification that remains widely used today. In recent years, the application of plastome data has greatly improved the resolution and support values of phylogenetic trees. Xie et al. [36] analyzed 68 cp protein-coding genes from 41 species representing 11 tribes of Plantaginaceae, identifying three core clades within the family. In their analysis, Russelieae and Cheloneae were recovered as sister tribes with full (100%) bootstrap support, Plantagineae and Veroniceae appeared closely related, and Callitricheae diverged at the base of the family. Their study not only resolved controversies regarding the phylogenetic positions of several tribes but also validated the scientific basis of “molecular evidence-based tribal division” in the APG system. Additionally, combined analyses of nuclear internal transcribed spacer (ITS) sequences and cp genes further consolidated the monophyly of Plantaginaceae and the relationships among its tribes.

*Penstemon* (beardtongue) is a core genus in the tribe Cheloneae of Plantaginaceae, and its monophyly has long been controversial [64]. Early morphology-based classifications suggested it might be polyphyletic due to overlapping characteristics with related genera such as *Russelia* and *Chelone*. While studies based on ITS sequences and a few cp genes (e.g., *trnS-trnG*, *rps16*) initially supported its monophyly, some nodes lacked sufficient support. However, Stettler et al. [35] sequenced plastomes of six *Penstemon* species, and Xie et al. [36] analyzed four *Penstemon* species in their inter-tribal study. Both analyses recovered the sampled *Penstemon* species as a well-supported monophyletic clade that was sister to *Russelia*. These findings provided strong support for the monophyly of the sampled species of *Penstemon*. However, because these analyses still cover only a small fraction of the approximately 280 described species, the genus-wide generality of this conclusion remains to be verified with broader taxon sampling.

In this study, we present the first fully characterized complete cp genome of *P. digitalis*. Our assembly and annotation confirmed a conserved quadripartite structure, with gene content and organization consistent with the typical features of the tribe Cheloneae in Plantaginaceae and highly similar to those of related genera such as Russelia. Comparative analyses identified several hypervariable regions and repetitive sequences that hold promise as candidate molecular markers for future population genetic studies and species delimitation in *Penstemon*. Furthermore, our phylogenetic reconstruction based on the complete cp genome recovered *Penstemon* as monophyletic with 100% bootstrap support in the present sampling and supported a sister relationship between *Penstemon* and *Russelia*, consistent with previous studies [35,36]. Beyond adding a horticulturally and medicinally important species to the list of sequenced *Penstemon* plastomes, this work provides an updated inventory of candidate hypervariable markers for the tribe Cheloneae. Nevertheless, as plastome data are currently available for only eight of the approximately 280 described species, our phylogenetic conclusions should be read as applying to the taxa sampled to date rather than to the genus as a whole. Notably, the complete plastome captures more evolutionary signal than individual markers, thereby improving the resolution of interspecific relationships and offering new insights into the long-standing taxonomic controversies in *Penstemon* arising from morphological convergence and hybridization.

Despite these advances, significant sampling limitations remain. Although *Penstemon* comprises approximately 280 described species, plastome data are available for only eight species, including *P. digitalis* (accounting for less than 3% of the total). Existing studies have substantial sampling gaps: they do not adequately represent all subgenera and sections, and they often fail to include peripheral populations or morphologically hybrid groups. These gaps have resulted in unclear species boundaries and unresolved interspecific relationships within the genus. To address these issues, future research should expand sampling efforts to systematically sequence plastomes of representative species from different evolutionary lineages and key taxa of *Penstemon*, integrating these data with nuclear genome sequences for combined analyses. Moreover, the candidate molecular markers identified in this study, once experimentally validated, could be used to investigate population-level genetic diversity and gene flow in widely distributed species, facilitating accurate species delimitation and clarifying the impacts of hybridization and adaptive radiation on speciation. Such an integrated approach will provide a more comprehensive theoretical basis for taxonomic revision, resource conservation, and sustainable utilization of *Penstemon*.

## 5. Conclusions

In this study, we characterized the first complete cp genome of *P. digitalis*, revealing a conserved quadripartite structure with gene content and organization typical of the tribe Cheloneae in Plantaginaceae. Comparative genomic analyses identified hypervariable regions and repetitive sequences that hold potential as candidate molecular markers for future population genetic studies and species delimitation in *Penstemon*. Phylogenetic reconstruction based on the complete cp genome recovered *Penstemon* as monophyletic and supported a sister relationship with *Russelia* in the present sampling, consistent with previous studies. Our findings enrich the genomic resources for this ecologically and horticulturally valuable genus and provide a foundation for resolving long-standing taxonomic controversies and advancing studies on population genetics, adaptive radiation, and conservation of *Penstemon*.

## Figures and Tables

**Table 1:** Gene function statistics table of chloroplast genome in *P. digitalis*.

Category	Group of Genes	Name of Genes
Self-replication	Large subunit of ribosome	*rpl20*, *rpl22*, *rpl32*, *rpl23(2)*, *rpl14*, *rpl33*, rpl16*, *rpl36*, rpl2*(2)
	Small subunit of ribosome	*rps11*, *rps14*, *rps15*, rps16*, *rps2*, *rps3*, *rps18*, *rps4*, *rps19*, *rps7(2)*, *rps8*, rps12**(2)
	DNA dependent RNA polymerase	*rpoA*, *rpoB*, rpoC1*, *rpoC2*
	rRNA gene	*rrn5(2)*, *rrn4.5(2)*, *rrn16(2)*, *rrn23(2)*
	tRNA gene	*trnR-UCU*, *trnM-AUG*, *trnE-UUC*, *trnT-GGU*, *trnS-GGA*, *trnV-GAC(2)*, *trnR-ACG(2)*, trnL-UAA*, *trnG-GCC*, *trnD-GUC*, *trnY-GUA*, *trnP-UGG*, *trnL-CAA(2)*, *trnM-CAU*, *trnS-GCU*, *trnW-CCA*, *trnF-GAA*, *trnT-UGU*, *trnS-UGA*, trnV-UAC*, trnG-UCC*, *trnL-UAG*, trnI-GAU*(2), *trnH-GUG*, *trnH-CAU(2)*, *trnQ-UUG*, *trnN-GUU(2)*, trnK-UUU*, trnA-UGC*(2), *trnC-GCA*
Gene for photosynthesis	Subunits of photosystem I	*psaA*, *psaB*, *psaC*, *psaI*, *psaJ*
	Subunits of photosystem II	*psbL*, *psbZ*, *psbM*, *psbN*, *psbA*, *psbB*, *psbC*, *psbD*, *psbE*, *psbF*, *psbT*, *psbH*, *psbI*, *psbJ*, *psbK*
	Subunits of NADH-dehydrogenase	*ndhG*, *ndhH*, *ndhI*, *ndhJ*, *ndhK*, ndhA*, ndhB*(2), *ndhC*, *ndhD*, *ndhE*, *ndhF*
	Subunits of cytochrome b/f complex	*petL*, *petN*, *petA*, petB*, petD*, *petG*
	Subunit for ATP synthase	*atpI*, *atpA*, *atpB*, *atpE*, atpF*, *atpH*
	Large subunit of rubisco	*rbcL*
Other genes	Translational initiation factor	*infA*
	Maturase	*matK*
	Protease	clpP**
	Envelope membrane protein	*cemA*
	Subunit of Acetyl-carboxylase	*accD*
	C-type cytochrome synthesis gene	*ccsA*
	Open reading frames (ORF, *ycf*)	*ycf1*, *ycf2(2)*, ycf3**, *ycf4*, *ycf15(2)*

Notes: Gene *: Gene with one intron and two exons; Gene **: Gene with two introns and three exons; Gene (2): Number of copies of multi-copy genes. Full gene names are listed in Table S2.

## Data Availability

The data that support the findings of this study are openly available in GenBank of NCBI at https://www.ncbi.nlm.nih.gov/ (accessed on 10 January 2024). The complete cp genome has been deposited in GenBank under the accession no. NC_086665.1. The associated Bioproject, SRA, Biosample numbers are PRJNA1064432, SRX23207097, and SAMN39432887 respectively.

## Supplementary Materials

The following supporting information can be downloaded at https://www.mdpi.com/article/10.3390/genes17101271/s1, Figure S1: Sequencing depth and coverage map of the *P. digitalis* chloroplast genome; Table S1: RSCU analysis of each amino acid in *P. digitalis*; Table S2: Full names of the gene abbreviations used in Table 1.

## Abbreviations

The following abbreviations are used in this manuscript:

cp	Chloroplast
ML	Maximum likelihood
PGA	Plastid Genome Annotator
NCBI	National Center for Biotechnology Information
SSRs	Simple sequence repeats
MISA	MIcroSAtellite
RSCU	Relative synonymous codon usage
MAFFT	Multiple Alignment using Fast Fourier Transform
Pi	Nucleotide diversity
IR	Inverted repeat
LSC	Large single-copy region
SSC	Small single-copy region
PCGs	Protein-coding genes
APG	Angiosperm Phylogeny Group
ITS	Internal transcribed spacer
