# Supplementary material for: Comparative Analysis of Complete Chloroplast Genomes of Penstemon digitalis Nutt. ex Sims and Insights into Phylogenetic Relationships of Penstemon
Source: Genes (Basel). 2026 Oct 13;17(10):1271. doi: 10.3390/genes17101271 (PMC13704105; doi:10.3390/genes17101271)
Supplement: Supplementary file 1 [file genes-17-01271-s001.zip › Supplemental Table S1.pdf]

Supplemental Table S1. RSCU analysis of each amino acid in *P. digitalis*

Amino acid	Codon	Number	RSCU	Amino acid	Codon	Number	RSCU
**Phe**	UUU	914	1.5	**His**	CAU	459	1.53
	UUC	473	0.5		CAC	137	0.36
**Leu**	UUA	795	2.07	**Gln**	CAA	686	1.58
	UUG	548	1.22		CAG	207	0.42
	CUU	575	1.28	**Ala**	GCU	553	1.77
	CUC	160	0.31		GCC	227	0.69
	CUA	369	0.83		GCA	369	1.12
	CUG	168	0.3		GCG	143	0.42
**Ile**	AUU	1011	1.52	**TER**	UGA	13	0.67
	AUC	442	0.58		UAA	32	1.66
	AUA	639	0.9		UAG	13	0.67
**Met**	AUG	562	1	**Trp**	UGG	436	0.88
**Val**	GUU	502	1.56	**Asn**	AAU	943	1.52
	GUC	155	0.39		AAC	278	0.45
	GUA	486	1.48	**Lys**	AAA	974	1.53
	GUG	178	0.57		AAG	345	0.44
**Ser**	UCU	547	1.63	**Arg**	CGU	310	1.55
	UCC	316	1		CGC	97	0.43
	UCA	394	1.19		CGA	349	1.53
	UCG	205	0.55		CGG	117	0.4
	AGU	382	1.27		AGA	452	1.61
	AGC	116	0.37		AGG	168	0.47
**Pro**	CCU	382	1.35	**Asp**	GAU	831	1.58
	CCC	207	0.76		GAC	195	0.42
	CCA	306	1.25	**Glu**	GAA	976	1.57
	CCG	142	0.56		GAG	319	0.43
**Thr**	ACU	500	1.51	**Gly**	GGU	522	1.26
	ACC	236	0.86		GGC	162	0.36
	ACA	380	1.22		GGA	678	1.66
	ACG	142	0.41		GGG	314	0.72
**Tyr**	UAU	740	1.64	**Cys**	UGU	205	1.47
	UAC	157	0.29		UGC	71	0.33
**Total**						24710	
