# Supplementary material for: Comparative Analysis of Complete Chloroplast Genomes of Penstemon digitalis Nutt. ex Sims and Insights into Phylogenetic Relationships of Penstemon
Source: Genes (Basel). 2026 Oct 13;17(10):1271. doi: 10.3390/genes17101271 (PMC13704105; doi:10.3390/genes17101271)
Supplement: Supplementary file 1 [file genes-17-01271-s001.zip › Supplemental Table S2.pdf]

Supplemental Table S2. Full names of the gene abbreviations used in Table 1.

Gene abbreviations follow the standard chloroplast genome nomenclature; full names are listed by functional category as in Table 1.

Gene abbreviation	Full name
**Self-replication — Large subunit of ribosome**	
rpl2	Ribosomal protein L2 of the 50S large subunit
rpl14	Ribosomal protein L14 of the 50S large subunit
rpl16	Ribosomal protein L16 of the 50S large subunit
rpl20	Ribosomal protein L20 of the 50S large subunit
rpl22	Ribosomal protein L22 of the 50S large subunit
rpl23	Ribosomal protein L23 of the 50S large subunit
rpl32	Ribosomal protein L32 of the 50S large subunit
rpl33	Ribosomal protein L33 of the 50S large subunit
rpl36	Ribosomal protein L36 of the 50S large subunit
**Self-replication — Small subunit of ribosome**	
rps2	Ribosomal protein S2 of the 30S small subunit
rps3	Ribosomal protein S3 of the 30S small subunit
rps4	Ribosomal protein S4 of the 30S small subunit
rps7	Ribosomal protein S7 of the 30S small subunit
rps8	Ribosomal protein S8 of the 30S small subunit
rps11	Ribosomal protein S11 of the 30S small subunit
rps12	Ribosomal protein S12 of the 30S small subunit
rps14	Ribosomal protein S14 of the 30S small subunit
rps15	Ribosomal protein S15 of the 30S small subunit
rps16	Ribosomal protein S16 of the 30S small subunit
rps18	Ribosomal protein S18 of the 30S small subunit
rps19	Ribosomal protein S19 of the 30S small subunit
**Self-replication — DNA-dependent RNA polymerase**	
rpoA	DNA-directed RNA polymerase subunit alpha
rpoB	DNA-directed RNA polymerase subunit beta
rpoC1	DNA-directed RNA polymerase subunit beta'
rpoC2	DNA-directed RNA polymerase subunit beta''
**Self-replication — rRNA genes**	
rrn4.5	4.5S ribosomal RNA
rrn5	5S ribosomal RNA
rrn16	16S ribosomal RNA
rrn23	23S ribosomal RNA
**Self-replication — tRNA genes**	
trnR-UCU	Transfer RNA for Arginine with anticodon UCU

Gene abbreviation	Full name
trnM-AUG	Transfer RNA for Methionine with anticodon AUG
trnE-UUC	Transfer RNA for Glutamic acid with anticodon UUC
trnT-GGU	Transfer RNA for Threonine with anticodon GGU
trnS-GGA	Transfer RNA for Serine with anticodon GGA
trnV-GAC	Transfer RNA for Valine with anticodon GAC
trnR-ACG	Transfer RNA for Arginine with anticodon ACG
trnL-UAA	Transfer RNA for Leucine with anticodon UAA
trnG-GCC	Transfer RNA for Glycine with anticodon GCC
trnD-GUC	Transfer RNA for Aspartic acid with anticodon GUC
trnY-GUA	Transfer RNA for Tyrosine with anticodon GUA
trnP-UGG	Transfer RNA for Proline with anticodon UGG
trnL-CAA	Transfer RNA for Leucine with anticodon CAA
trnM-CAU	Transfer RNA for Methionine with anticodon CAU
trnS-GCU	Transfer RNA for Serine with anticodon GCU
trnW-CCA	Transfer RNA for Tryptophan with anticodon CCA
trnF-GAA	Transfer RNA for Phenylalanine with anticodon GAA
trnT-UGU	Transfer RNA for Threonine with anticodon UGU
trnS-UGA	Transfer RNA for Serine with anticodon UGA
trnV-UAC	Transfer RNA for Valine with anticodon UAC
trnG-UCC	Transfer RNA for Glycine with anticodon UCC
trnL-UAG	Transfer RNA for Leucine with anticodon UAG
trnI-GAU	Transfer RNA for Isoleucine with anticodon GAU
trnH-GUG	Transfer RNA for Histidine with anticodon GUG
trnH-CAU	Transfer RNA for Histidine with anticodon CAU
trnQ-UUG	Transfer RNA for Glutamine with anticodon UUG
trnN-GUU	Transfer RNA for Asparagine with anticodon GUU
trnK-UUU	Transfer RNA for Lysine with anticodon UUU
trnA-UGC	Transfer RNA for Alanine with anticodon UGC
trnC-GCA	Transfer RNA for Cysteine with anticodon GCA
**Photosynthesis — Subunits of Photosystem I**	
psaA	Photosystem I P700 chlorophyll a apoprotein A1
psaB	Photosystem I P700 chlorophyll a apoprotein A2
psaC	Photosystem I iron-sulfur center subunit VII
psaI	Photosystem I reaction center subunit VIII
psaJ	Photosystem I reaction center subunit IX
**Photosynthesis — Subunits of Photosystem II**	
psbA	Photosystem II D1 reaction center protein
psbB	Photosystem II CP47 chlorophyll apoprotein

Gene abbreviation	Full name
psbC	Photosystem II CP43 chlorophyll apoprotein
psbD	Photosystem II D2 reaction center protein
psbE	Photosystem II cytochrome b559 subunit alpha
psbF	Photosystem II cytochrome b559 subunit beta
psbH	Photosystem II 10 kDa phosphoprotein
psbI	Photosystem II reaction center I protein
psbJ	Photosystem II reaction center J protein
psbK	Photosystem II reaction center K protein
psbL	Photosystem II reaction center L protein
psbM	Photosystem II reaction center M protein
psbN	Photosystem II reaction center N protein
psbT	Photosystem II reaction center T protein
psbZ	Photosystem II reaction center Z protein
**Photosynthesis — Subunits of NADH dehydrogenase**	
ndhA	NADH-plastoquinone oxidoreductase subunit 1
ndhB	NADH-plastoquinone oxidoreductase subunit 2
ndhC	NADH-plastoquinone oxidoreductase subunit 3
ndhD	NADH-plastoquinone oxidoreductase subunit 4
ndhE	NADH-plastoquinone oxidoreductase subunit 4L
ndhF	NADH-plastoquinone oxidoreductase subunit 5
ndhG	NADH-plastoquinone oxidoreductase subunit 6
ndhH	NADH-plastoquinone oxidoreductase subunit 7
ndhI	NADH-plastoquinone oxidoreductase subunit 8
ndhJ	NADH-plastoquinone oxidoreductase subunit 9
ndhK	NADH-plastoquinone oxidoreductase subunit 10
**Photosynthesis — Subunits of Cytochrome b/f complex**	
petA	Cytochrome f apoprotein of the cytochrome b6/f complex
petB	Cytochrome b6 subunit of the cytochrome b6/f complex
petD	Subunit IV of the cytochrome b6/f complex
petG	PetG subunit of the cytochrome b6/f complex
petL	PetL subunit of the cytochrome b6/f complex
petN	PetN subunit of the cytochrome b6/f complex
**Photosynthesis — Subunits for ATP synthase**	
atpA	ATP synthase subunit alpha (CF1)
atpB	ATP synthase subunit beta (CF1)
atpE	ATP synthase subunit epsilon (CF1)
atpF	ATP synthase subunit I (CF0)
atpH	ATP synthase subunit III (CF0)

Gene abbreviation	Full name
atpI	ATP synthase subunit IV (CF0)
**Photosynthesis — Large subunit of Rubisco**	
rbcL	Large subunit of ribulose-1,5-bisphosphate carboxylase/oxygenase
**Other genes — Translational initiation factor**	
infA	Translation initiation factor 1
**Other genes — Maturase**	
matK	Maturase K
**Other genes — Protease**	
clpP	ATP-dependent Clp protease proteolytic subunit
**Other genes — Envelope membrane protein**	
cemA	Chloroplast envelope membrane protein A
**Other genes — Subunit of Acetyl-carboxylase**	
accD	Acetyl-CoA carboxylase carboxyl transferase subunit beta
**Other genes — C-type cytochrome synthesis gene**	
ccsA	C-type cytochrome synthesis protein A (heme delivery protein)
**Open reading frames (ORF, *ycf*)**	
ycf1	Hypothetical chloroplast open reading frame 1
ycf2	Hypothetical chloroplast open reading frame 2
ycf3	Hypothetical chloroplast open reading frame 3
ycf4	Hypothetical chloroplast open reading frame 4
ycf15	Hypothetical chloroplast open reading frame 15
